# Fatty Acid and Lipid Metabolism in Oil Palm: From Biochemistry to Molecular Mechanisms

**DOI:** 10.3390/ijms26062531

**Published:** 2025-03-12

**Authors:** Eman H. Afifi, Jerome Jeyakumar John Martin, Qi Wang, Xinyu Li, Xiaoyu Liu, Lixia Zhou, Rui Li, Dengqiang Fu, Qihong Li, Jianqiu Ye, Hongxing Cao

**Affiliations:** 1 National Key Laboratory for Tropical Crop Breeding, Chinese Academy of Tropical Agricultural Sciences, Haikou 571101, China; emanheay@gmail.com (E.H.A.); jeromejeyakumarj@gmail.com (J.J.J.M.); 12023131688@stu.nxu.edu.cn (Q.W.); lixinyu@catas.cn (X.L.); liuxy86@catas.cn (X.L.); lxzhou@catas.cn (L.Z.); lirui@catas.cn (R.L.); fudq@catas.cn (D.F.); liqihong@catas.cn (Q.L.); 2 Coconut Research Institute, Chinese Academy of Tropical Agricultural Sciences, Wenchang 571339, China; 3 Agriculture Research Center, Horticulture Research Institute, Fruit Breeding Department, Giza 12619, Egypt

**Keywords:** oil palm, fatty acid metabolism, lipid biosynthesis, lipid accumulation, biochemical pathways, molecular mechanisms

## Abstract

Oil palm (*Elaeis guineensis*) is a cornerstone of the economy in many countries due to its unparalleled ability to produce high yields of oil, making it a critical crop among oil-producing fruits. This review aims to elucidate the processes involved in fatty acid formation and synthesis, which are essential components of palm oil, and to examine the changes these fatty acids undergo during fruit growth and ripening. Additionally, we highlight the genes and molecular mechanisms governing fatty acid metabolism, which hold significant potential for influencing oil composition and quality. Understanding these pathways is vital, as fatty acid profiles have profound implications for both human health and industrial applications. While palm oil contains beneficial compounds, such as polyphenols and vitamin E, concerns arise from its high palmitic acid content and the formation of potentially harmful byproducts during industrial refining, such as 3-monochloropropane-1,2-diol (3-MCPD) esters and glycidyl esters. We also explore advanced breeding methods and modern strategies to enhance oil quality and productivity, including the application of genomic tools to transfer desirable traits and expand genetic diversity in breeding programs. By integrating biochemical, genetic, and biotechnological insights, this review provides a foundation for improving palm oil production and addressing the growing demand for healthier, sustainable oil solutions.

## 1. Introduction

**Figure 1:**
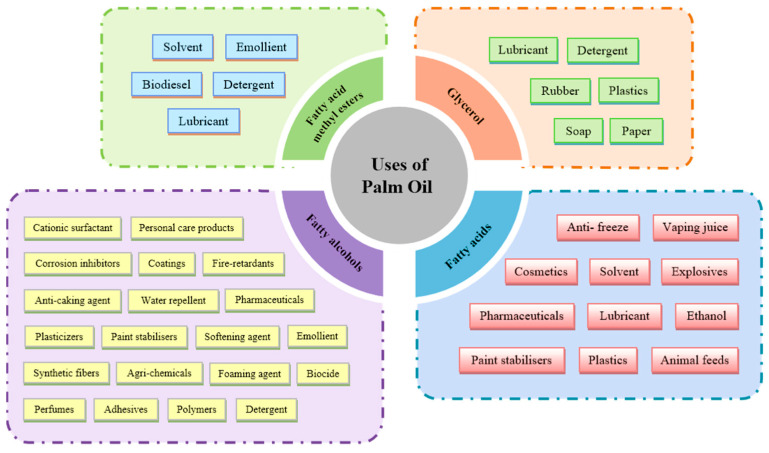
Importance and uses of palm oil.

Oil palm (*Elaeis guineensis*) is a vital crop globally, contributing significantly to the production of vegetable oils used for food, industrial applications, and biofuels. With its high oil yield per hectare, oil palm is central to the economies of many tropical regions, providing livelihoods for millions of people [1,2]. The oil derived from its fruits is rich in fatty acids, which form the primary components of palm oil, and its composition is a critical determinant of oil quality and functionality [3,4]. Fatty acid and lipid metabolism in oil palm are complex processes that involve multiple biochemical pathways and regulatory mechanisms. These processes govern the synthesis, elongation, desaturation, and storage of fatty acids, ultimately influencing the yield, composition, and nutritional value of the oil [5]. Understanding these pathways is crucial for optimizing oil palm productivity, improving oil quality, and addressing challenges such as increasing global demand, sustainability concerns, and the impact of environmental stresses, including cold temperatures [6]. Palm oil is a widely used vegetable oil, essential in various industries due to its unique properties and versatility (Figure 1). Palm oil is widely used due to its versatility and economic value, playing a key role in the food, cosmetics, and pharmaceutical industries. It naturally contains beneficial compounds, such as free fatty acids, polyphenols, and vitamin E, which may offer health advantages when consumed in unrefined or minimally processed forms. However, concerns have been raised regarding the health implications of its consumption, particularly in its refined and processed forms. Industrial refining can lead to the formation of compounds such as 3-monochloropropane-1,2-diol (3-MCPD) esters and glycidyl esters (GE), which have been classified as potential health risks by regulatory bodies, including the European Food Safety Authority (EFSA) and the World Health Organization (WHO) [7,8]. Additionally, palm oil’s high palmitic acid content has been associated with increased blood cholesterol levels, leading to recommendations to limit saturated fat intake [9]. Despite these concerns, palm oil remains an important global commodity, and efforts to improve its nutritional profile—such as increasing the proportion of monounsaturated fats like oleic acid—have gained attention in breeding and genetic engineering programs.

Beyond health considerations, palm oil production has also faced scrutiny for its environmental impact, particularly deforestation, habitat loss, and biodiversity decline. However, initiatives like the Roundtable on Sustainable Palm Oil (RSPO) aim to promote responsible production practices. The palm oil industry remains a major contributor to the economies of producing countries, especially in Southeast Asia, where it drives employment and economic growth while also addressing global food and industrial needs. Advances in molecular biology and omics technologies have provided new insights into the genes, enzymes, and regulatory networks involved in fatty acid metabolism [7,8]. These discoveries enable targeted breeding programs and biotechnological interventions aimed at improving oil composition and yield. Additionally, they offer opportunities to address human health concerns associated with excessive consumption of industrially processed oils by tailoring fatty acid profiles for healthier dietary options [9,10].

This review focuses on the biochemical and molecular mechanisms underlying fatty acid and lipid metabolism in oil palm. It examines the key enzymes and regulatory genes involved, the changes in lipid composition during fruit development, and the influence of environmental factors on lipid biosynthesis [2,8]. Furthermore, the review explores modern breeding approaches and genetic engineering strategies for enhancing oil palm productivity and sustainability. By synthesizing current knowledge, this study aims to provide a comprehensive framework for future research and practical applications in oil palm improvement.

## 2. Fatty Acid Synthesis and Accumulation in Oil Palm

**Figure 2:**
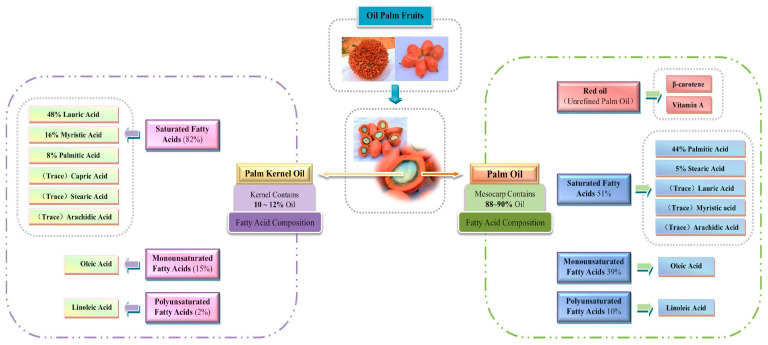
The percentage of saturated and unsaturated fats in oil and their different forms in palm oil.

The mesocarp of oil palms accumulates a remarkably high amount of oil, indicating the presence of a distinct transcriptional network for fatty acid metabolism specific to the fruit [11]. Two highly valuable oils are extracted from the mesocarp and endosperm, respectively, known as palm oil and palm kernel oil. Palmitic and oleic acids are the main fatty acids (FAs) in mesocarp oil (Figure 2), while lauric acid is the predominant FA in endosperm oil (Table 1). Additionally, the oil palm embryo stores oil, which has a high linoleic acid content [12]. Free Fatty Acid (FFA) is a criterion used to assess the quality of crude palm oil (CPO), with a standard value of less than 5%. High FFA in CPO leads to low refining rates and quality, such as rancidity and odor. Any increase in the FFA value should be avoided with proper fruit handling and appropriate maturity levels. Even with proper handling, the natural process of delayed delivery to the processing facility after harvesting will raise the FFA value [13].

Mesocarp metabolites were profiled during six critical phases of fruit development in populations of oil palms with relatively high and low yields, using multi-platform metabolomics technology. A higher-yielding commercial palm population was found to have significantly higher levels of nucleosides during lipid biosynthesis and amino acids prior to lipid biosynthesis. An increase in the ratio of malic acid to citric acid demonstrated differences in carbon utilization in the TCA cycle, while metabolites involved in glycolysis showed an intriguing shift toward glycerol-3-phosphate [14]. Up-regulated genes involved in glycolysis, TCA, and fatty acid biosynthesis used a higher carbon flux (channeled through down-regulation of the Sucrose Synthase 2 pathway) to increase oil production [15] (Table 2). This illustrates how the availability of sugar, lipid synthesis rates, and metabolic recycling all affect oil yield [16].

Proteomics and metabolomics also offer methods to explore lipid creation and stress responses, control fatty acid composition through various gene and metabolite levels, understand physiological reactions to biotic stresses, and clarify biological processes in oil palm [21]. Recently, iTRAQ labeling in conjunction with 2D-LC and MALDI-TOF/TOF MS, was used to analyze the proteomic profiles of oil palm mesocarps at four developmental stages: 12, 16, 18, and 22 weeks post-pollination. It was discovered that there was coordinated differential expression of proteins involved in key metabolic processes, including fatty acid biosynthesis. This increase in protein expression resulted in higher carbon flux, and resources needed for lipid biosynthesis, like ATP and NADH, were redirected [22].

Similarly, LC-MS/MS was used to analyze free fatty acid content and composition in oil palm flesh at three different time points: 95 days (MS1 and MT1), 125 days (MS2 and MT2), and 185 days (MS3 and MT3) after pollination of Seedless (MS) and Tenera (MT) oil palm fruit species. RNA-Seq was employed to analyze gene expression related to free fatty acid synthesis and accumulation. Variations in genes and metabolites were linked to the KEGG (Kyoto Encyclopedia of Genes and Genomes) pathway map through KEGG enrichment analysis. A metabolomics study found 17 saturated and 13 unsaturated free fatty acids during the development of MS and MT. Significant variations in gene expression were found in transcriptomic research between the three MT and MP harvest phases [23].

In the mesocarp and kernel tissues of mature oil palm fruits, fatty acid phytyl esters (FAPEs) were found, primarily esterified with oleic acid (C18:1) and very long-chain fatty acids (VLCFAs), such as arachidic acid (C20:0), behenic acid (C22:0), and lignoceric acid (C24:0), which are present in lower proportions compared to palmitic and oleic acids. The quantity and composition of FAPEs and FAGGEs in mesocarp and kernel tissues varied significantly among Cameroonian wild accessions of the African oil palm. As a result, these compounds are considered an additional metabolic source for phytol or geranylgeraniol and fatty acids, respectively [24]. Furthermore, the high tocochromanol content and extremely low palmitate content of Cote d’Ivoire populations distinguish them from populations of other origins. High levels of carotene were found in genotypes from Nigeria, Cote d’Ivoire, and Benin [25].

## 3. Regulation of Lipid Biosynthesis and Accumulation in Oil Palm

Lauric acid accumulation in oil palm is dependent on the coordinated recruitment of specific isoforms of triacylglycerol assembly enzymes, as well as the up-regulation of a specialized acyl–acyl carrier protein thioesterase paralog [8]. Targeted lipidomic analysis of oil palm reveals a wide range of molecular species within each lipid class, with the majority of lipid accumulation occurring during the middle and final stages of storage. These studies highlight notable variations in lipid biosynthesis and accumulation patterns during different phases of fruit development [26].

Triacylglycerol (TAG) synthesis in the endoplasmic reticulum is regulated by key target genes. In particular, β-ketoacyl-CoA synthase 12 (KCS12) is targeted by the microRNAs Eg-miR156e and Eg-miR156j, while very-long-chain enoyl-CoA reductase (ECR) is targeted by nov-miR201. It is also predicted that several microRNAs indirectly control lipid metabolism and fatty acid (FA) synthesis by influencing transcription factors such as MYB, NAC, and *squamosa promoter-binding protein-like genes* (SPL) [27]. Additionally, diacylglycerol acyltransferase (DGAT) plays a crucial role in TAG accumulation in the oil palm mesocarp. Electrophoretic mobility shift assays (EMSA) and dual-luciferase reporter gene assays confirmed that the transcription factor EgbHLH63 binds to the promoter of EgDGAT1. Overexpression of EgbHLH63 in oil palm protoplasts led to a significant reduction in EgDGAT1 expression, while silencing EgbHLH63 in transgenic embryoids resulted in higher TAG content and restored high EgDGAT1 expression. Other transcription factors, such as EgULT1 and EgZFP, were also found to interact with EgbHLH63. Motif analysis identified AT-rich and B-box motifs in the oil palm genome [28].

Temperature also affects lipid synthesis in oil palm. Studies using tissue cultures have shown that lipid synthesis increases when the temperature is raised from 20 °C to 30 °C. While total lipid labeling almost doubled under higher temperatures, there was no significant change in the labeling of intermediate acyl-(acyl carrier protein) or acyl-CoA pools. Interestingly, TAG labeling increased while polyunsaturated fatty acid labeling decreased at elevated temperatures. However, there was no increase in the labeling of the intermediate diacylglycerol pool. These findings suggest that temperature plays a significant role in regulating lipid biosynthesis in oil palm [29,30]. In the oil palm kernel and shell tissues, proteins associated with storage and TAG synthesis were more prevalent. Enzymes involved in stilbenoid biosynthesis, such as trans-resveratrol di-O-methyltransferase (ROMT), hydroxycinnamoyl-CoA: shikimate hydroxycinnamoyl transferase (HCT), and 4-coumarate: coenzyme A ligase (4CL), were highly expressed in the exocarp, mesocarp, and shell tissues but not in the kernel [31].

To gain a deeper understanding of lipid biosynthesis, palm metabolites were profiled during six critical stages of fruit development using multiplatform metabolomics technology. Prior to lipid biosynthesis, the palm mesocarp exhibited significantly higher levels of amino acids. Metabolites involved in the tricarboxylic acid cycle were more abundant during early fruit development, while nucleosides were found to be in high concentration during lipid biosynthesis [32]. In oil-producing species, oil synthesis is closely linked to starch metabolism. High glycolytic activity, rapid starch turnover, and sucrose flux to the mesocarp tissue are essential for oil production in oil palms [33]. Additionally, oil yield categories varied significantly in terms of starch granule morphology, granule size, total starch content, and starch chain length distribution (CLD), with several starch parameters strongly correlated with oil yield. These findings also indicate that lipid and carotenoid biosynthesis in oil palm monocot fruits has diverged from that of dicot fruits [7]. During fruit development, the most drastically altered proteins are those involved in lipid synthesis, energy production, secondary metabolite formation, and amino acid metabolism. These proteins represent potential targets for enhancing oil yield in oil palm [34].

## 4. Molecular Mechanisms of Fatty Acid Synthesis and Oil Accumulation in Oil Palm

**Figure 3:**
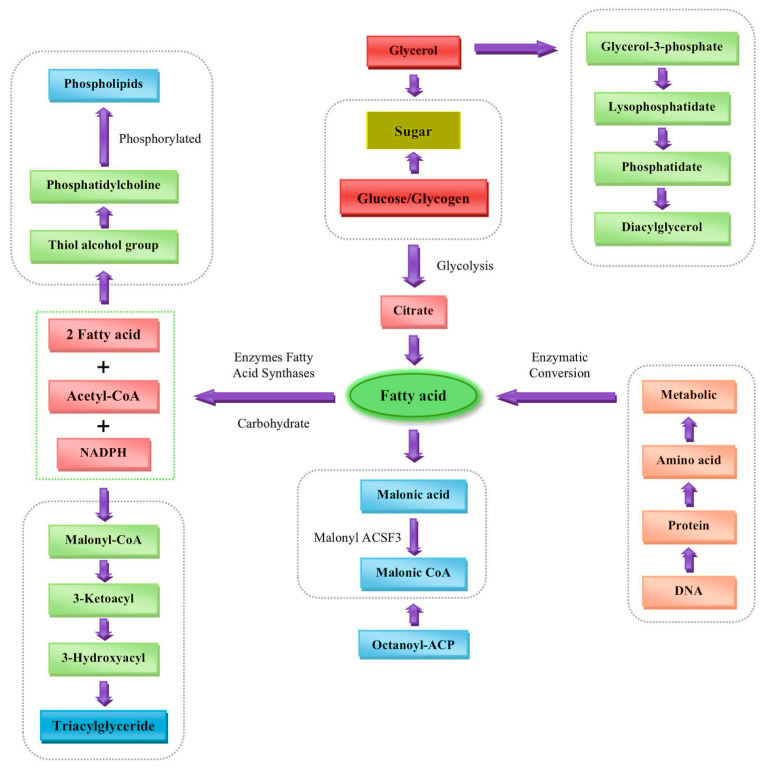
Fatty acid synthesis.

Recent advancements in oil palm research have highlighted the significant role of histological techniques, genome sequencing, and transcriptomics in advancing our understanding of the plant’s biology. The sequencing of the complete oil palm genome has provided insights into its genetic structure, aiding in the creation of molecular markers and genetic maps essential for studying key traits and genetic materials in oil palm. Transcriptomics has become a valuable tool for investigating various biological aspects, including biotic and abiotic stress responses, fatty acid synthesis (Figure 3) and accumulation, and sexual reproduction in oil palm [17].

Research has shown that the high expression of certain MADS-box genes in the mesocarp of oil palm at various developmental stages suggests their critical role in cell division and metabolite accumulation. These genes are considered potential targets for studies on fruit development and oil accumulation [35]. Transcriptomic studies revealed 10,804 genes with significantly different expression between Seedless (MS) and Tenera (MT) oil palm varieties. Notably, the study found a negative correlation between FabB and the levels of free palmitic acid in the flesh of both MS and MT varieties, as well as a positive correlation with stearic acid, myristic acid, and palmitic acid levels. The expression of genes such as ACSL and FATB was positively correlated with these fatty acids, while FabB and FabF may regulate the synthesis of free myristate and palmitoleic acid, providing insights into enhancing the quality of palm oil [16].

A comprehensive analysis of 452 microRNAs (miRNAs), including 170 known and 282 novel miRNAs, identified potential regulators of fatty acid metabolism in the oil palm mesocarp (Table 3). Twenty-two known miRNAs and fourteen novel miRNAs were predicted to target 37 genes involved in fatty acid synthesis. For example, eg-miR156c, eg-miR397, eg-miR444b, and nov-miR129 target key enzymes such as acetyl-CoA carboxylase (ACC1), long-chain acyl-CoA synthetase (LACS), and enoyl-ACP reductase (ENR) to regulate fatty acid synthesis in plastids and facilitate fatty acid transport to the endoplasmic reticulum. This discovery highlights the complex miRNA-mRNA regulatory network involved in fatty acid metabolism in oil palm [36].

The oil palm mesocarp also contains the cDNA sequence EgFAD6, which encodes a plastidial ω6 fatty acid desaturase, a key enzyme in linoleic acid biosynthesis. Further studies confirmed the presence of another desaturase, FAD12, which contributes to the synthesis of linoleic acid [36,45]. Additionally, ChIP-seq and bioinformatics analyses identified potential downstream regulatory genes in the mesocarp, including EgAGL9, a transcription factor resembling MADS-box genes. This study confirmed the regulatory role of EgAGL9 in oil metabolism, with genes such as EgSAD, EgTSA, and EgSDH being directly influenced by it [46].

In protoplast studies, it was observed that EgFATA expression is up-regulated in response to transient overexpression of EgGRP2A, while its expression is significantly reduced when EgGRP2A is downregulated in transgenic oil palm embryoids [47,48]. Moreover, the paralogs EgWRI1-1 and EgWRI1-2, which are transcription factors related to WRINKLED1 (WRI1), were highly transcribed during oil deposition in the mesocarp and endosperm. These transcription factors are correlated with oil content, indicating their important role in fatty acid synthesis [49]. Research has also revealed that EgMADS3 regulates the biosynthesis of medium-chain fatty acids (MCFA) by positively influencing the expression of EgLPAAT, enhancing lipid content and MCFA accumulation. Overexpression of EgMYB108 in oil palm embryoids led to increased accumulation of very long-chain fatty acids (VLCFA) and total lipid content, further enhancing oil palm oil yield [8]. In conclusion, these findings contribute to a deeper understanding of the molecular mechanisms driving oil accumulation and fatty acid synthesis in oil palm. This knowledge is crucial for the development of effective breeding strategies and biotechnological approaches aimed at improving oil palm yields and oil quality [39].

## 5. Advances in Oil Palm Breeding: Leveraging Biotechnology and Molecular Tools for Enhanced Yields

**Figure 4:**
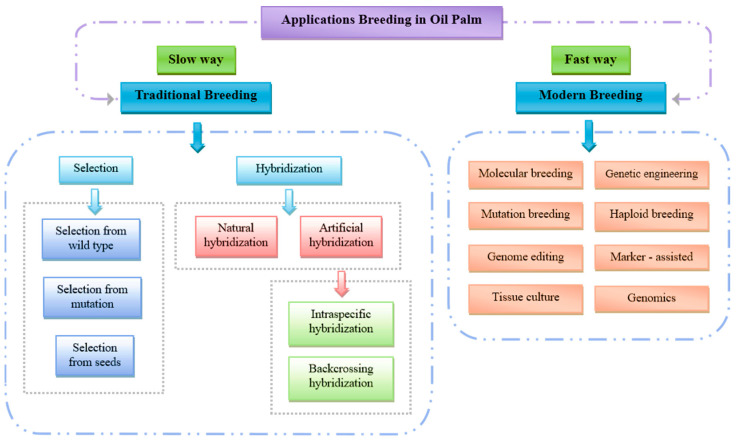
Various applications in the field of breeding and used in improving the quality of the oil palm.

Breeding programs are essential for producing superior oil palm planting materials to enhance yields (Figure 4). Traditional plant breeding has been a cornerstone of improving oil palm yield, with increases of 10–20% per generation. However, these programs are time-consuming, requiring 10–20 years for phenotypic selection [50]. Although traditional breeding plays a crucial role in boosting oil yields, the long breeding cycle (9–15 years) and the limited genetic pool have hindered progress in trait improvement [51]. A breakthrough came with the discovery of single gene inheritance for shell thickness (Sh gene), which significantly impacted the oil palm industry. Despite the challenges posed by lengthy breeding cycles, traditional breeding remains vital for addressing many issues, as long as there is sufficient genetic diversity available for breeders [52,53]. As oil palm germplasm is genetically characterized and beneficial traits are screened, breeders can leverage these resources for molecular transgenic breeding and marker-assisted selection.

Biotechnology has overcome the limitations of conventional breeding by enabling the isolation of genes and promoters, the creation of transformation vectors, and the successful transformation of genes into oil palm. Over the past 25 years, advancements in oil palm genetic transformation, such as particle bombardment and Agrobacterium-mediated methods, have made significant strides [48,54]. Biochemical–omics approaches, including transcriptomics, proteomics, and metabolomics, have also contributed to plant breeding efforts aimed at improving yields. These techniques allow researchers to target specific genes and regulatory elements to develop desired traits in oil palms.

In another study, a cross between the LM2T × DA10D oil palm genotypes and a pseudo-backcross with *E. oleifera* was examined. The analysis revealed that the proportions of FA and yield traits in *E. guineensis* were not highly correlated, suggesting that these traits can be selected independently. Quantitative trait loci (QTL) analysis identified several QTLs for FA traits, such as palmitic acid (C16:0), stearic acid (C18:0), oleic acid (C18:1), and linoleic acid (C18:2), with different mapping populations showing unique QTL regions for each trait [55]. Crosses between Nigerian and Côte d’Ivoire genotypes produced hybrids with crude palm oil (CPO) containing low palmitate and high levels of provitamin A and vitamin E [56]. Additionally, the development of 153 F1 trees from a cross between Ghana Pisifera and Deli Dura has provided a valuable breeding population for further studies [57].

The use of molecular tools, such as CAPS markers, accelerates the breeding process by allowing farmers to select high-yielding Tenera sprouts early, saving time, land, and money [57]. Genome editing technologies, such as CRISPR/Cas9, provide a means to introduce desired traits into oil palm without losing beneficial traits [58]. A linkage map with 1480 DNA markers has been used to identify QTLs for oil content traits, and marker densities have significantly improved the resolution of genetic mapping [59]. Furthermore, the use of whole-genome SNPs in breeding programs, particularly for dura, has shown increased genomic resolution and enhanced the efficiency of genomic selection (GS) for oil palm breeding [59].

Molecular breeding, incorporating advanced technologies such as tissue culture, haploid breeding, mutation breeding, marker-assisted selection (MAS), genomic selection (GS), and genome editing (GE), is expected to accelerate genetic improvements in oil palm. The development of low-lipase oil palm lines presents significant agronomic value, as these lines exhibit reduced enzymatic hydrolysis of triacylglycerols into free fatty acids (FFAs) during post-harvest storage. This reduction in FFA accumulation helps maintain oil quality, enhances oxidative stability, and extends shelf life, thereby improving economic efficiency and reducing processing costs in the palm oil industry [60].

## 6. Key Challenges in the Future of Palm Oil Production

Vegetable oils, including palm oil, make up a significant portion of the calories consumed globally. Among these oils, palm oil leads in terms of consumption and productivity, largely due to its favorable composition. However, the fatty acid composition of vegetable oils, including palm oil, does not fully meet the nutritional needs of humans or the demands of the food industry. Understanding the fundamental mechanisms behind the synthesis of both saturated and unsaturated fatty acids in palm oil is crucial. The quality of oil is primarily determined by its fatty acid profile, specifically the ratio of unsaturated to saturated fatty acids. This ratio is essential not only for oil quality but also for human health, as high intake of saturated fatty acids is linked to various diseases, particularly heart disease. Compared to palm oil, which has a high proportion of palmitic acid (C16:0), olive oil—especially extra virgin olive oil—is considered nutritionally superior due to its high oleic acid (C18:1) content, which has been associated with cardiovascular health benefits. To enhance the nutritional profile of palm oil, genetic modification and selective breeding strategies can be utilized to increase the proportion of oleic acid while reducing saturated fatty acids such as palmitic acid. This can be achieved through genetic engineering by downregulating genes involved in saturated fatty acid biosynthesis, such as those encoding stearoyl-ACP desaturase, or through conventional breeding by selecting oil palm varieties with naturally higher unsaturated fatty acid content. These efforts should focus on improving palm oil’s fatty acid composition to better align with both nutritional guidelines and industrial applications while maintaining its high yield and economic viability.

## 7. Conclusions

Oil palm remains one of the most economically and nutritionally valuable oil crops, yet several critical aspects require further exploration. While palm oil is highly productive and versatile, its fatty acid composition significantly influences its applications in the food, biofuel, and industrial sectors. The high content of saturated fats, particularly palmitic acid, makes it stable for cooking and food processing, but excessive consumption is linked to health concerns. On the other hand, modifying its fatty acid profile to increase unsaturated fats could enhance its nutritional value and expand its industrial applications, including bio-based lubricants and cosmetics. Future research should focus on comprehensively mapping fatty acid synthesis pathways and understanding how external factors—such as climate change, temperature, humidity, fertilization, and irrigation—impact oil quality. Additionally, comparative studies across different oil palm varieties can provide insights into genetic and metabolic variations that influence oil composition. Advances in genome sequencing, molecular breeding, and tissue culture techniques have facilitated progress in improving oil quality and sustainability. However, a deeper understanding of the genes, enzymes, and metabolic processes occurring within specific cellular compartments is necessary to optimize palm oil production for diverse applications while addressing both health and environmental concerns.

## Figures and Tables

**Table 1:** Fatty Acid Composition of Palm Oil (Different Cultivars and Growth Periods).

Cultivar	Growth Period	Palmitic Acid (C16:0)	Oleic Acid (C18:1)	Linoleic Acid (C18:2)	Stearic Acid (C18:0)	Other Fatty Acids
Tenera (Dura × Pisifera)	12 months	44.5%	39.2%	10.1%	4.5%	1.7%
Dura	12 months	47.3%	36.8%	9.8%	4.9%	1.2%
Pisifera	12 months	42.1%	41.5%	11.2%	3.8%	1.4%
Tenera (Nigeria)	18 months	43.8%	40.1%	10.5%	4.2%	1.4%
Tenera (Côte d’Ivoire)	18 months	42.9%	41.3%	10.8%	3.9%	1.1%
Tenera (Ghana)	24 months	41.5%	42.6%	11.0%	3.5%	1.4%
Deli Dura	24 months	46.8%	37.5%	9.5%	4.8%	1.4%

**Table 2:** Genes, Enzymes, and Their Roles in Fatty Acid Metabolism in Oil Palm.

Gene	Enzyme/Protein	Function	Role in Fatty Acid Metabolism	Reference
KAS I/II	3-Ketoacyl-ACP Synthase I/II	Elongates fatty acid chains during biosynthesis	Modulates elongation steps in fatty acid synthesis	[4]
KAS III	3-Ketoacyl-ACP Synthase III	Initiates fatty acid biosynthesis by condensing acetyl-CoA and malonyl-ACP	Regulates early stages of fatty acid biosynthesis by targeting KAS III transcripts	[17]
FATA/B	Acyl-ACP Thioesterase A/B	Terminates fatty acid synthesis by releasing free fatty acids	Influences the release of fatty acids from ACP	[8]
SAD	Stearoyl-ACP Desaturase	Converts stearic acid to oleic acid (introduces double bonds)	Regulates desaturation of fatty acids	[3]
FAD2	Fatty Acid Desaturase 2	Desaturates oleic acid to linoleic acid	Controls polyunsaturated fatty acid synthesis	[7]
DGAT1/2	Diacylglycerol Acyltransferase 1/2	Catalyzes the final step in triacylglycerol (TAG) biosynthesis	Regulates oil accumulation by targeting DGAT genes	[5]
PDAT	Phospholipid:DAG Acyltransferase	Alternative enzyme for TAG synthesis	Modulates TAG synthesis under stress conditions	[18]
ACP	Acyl Carrier Protein	Carries fatty acid chains during synthesis	Affects fatty acid chain elongation and transport	[19]
PEPC	Phosphoenolpyruvate Carboxylase	Provides carbon skeletons for fatty acid synthesis	Regulates carbon flux into fatty acid biosynthesis	[20]
WRI1	WRINKLED1 Transcription Factor	Regulates genes involved in fatty acid biosynthesis	Modulates expression of WRI1, affecting oil accumulation	[5]

**Table 3:** MicroRNAs (miRNAs) Involved in Lipid Metabolism: Target Genes/Proteins and Function.

miRNA	Target Genes/Proteins	Function in Lipid Metabolism	Reference
miR156	Squamosa Promoter-Binding Protein (SBP)	Regulates transcription factors, influencing lipid biosynthesis	[27]
miR159	MYB Transcription Factors	Modulates gene expression linked to fatty acid biosynthesis	[37]
miR167	Auxin Response Factor (ARF)	Influences fatty acid desaturation via hormonal regulation	[38]
miR172	APETALA2 (AP2)	Indirectly affects fatty acid synthesis pathways	[39]
miR319	TCP Transcription Factors	Regulates lipid metabolism and organ development	[40]
miR397	Laccase Genes	Impacts secondary metabolism, indirectly affecting lipid pathways	[41]
miR398	Cu/Zn Superoxide Dismutase (SOD)	Protects lipid membranes from oxidative damage	[42]
miR408	Plastocyanin-Like Proteins	Affects lipid metabolism under stress conditions	[43]
miR528	Acetyl-CoA Carboxylase (ACCase)	Regulates the first committed step in fatty acid biosynthesis	[44]

## Data Availability

Data are contained within the article.
